# Revealing asperity-controlled failure patterns in landslides: A case study of Hushuo Expressway, Inner Mongolia

**DOI:** 10.1371/journal.pone.0323903

**Published:** 2025-05-23

**Authors:** Xing Yuan-hao, Li Chi, Li Shuan-hu, Gao Yu

**Affiliations:** 1 College of Science, Inner Mongolia University of Technology, Hohhot, China; 2 Inner Mongolian Transportation Design & Research Institute Co., Ltd, Hohhot, China; 3 College of Resources and Environmental Engineering, Inner Mongolia University of Technology, Hohhot, China; 4 Key Laboratory of Geological Hazards and Geotechnical Engineering Defense in Sandy and Drought Regions at Universities of Inner Mongolia Autonomous Region, Inner Mongolia University of Technology, Hohhot, China; 5 Inner Mongolia Engineering Research Center of Geological Technology and Geotechnical Engineering, Inner Mongolia University of Technology, Hohhot, China; China Construction Fourth Engineering Division Corp. Ltd, CHINA

## Abstract

This study systematically investigates the failure mechanism of interfacial landslides through experimental validation and engineering applications of interfacial asperity theory. An innovative scaled physical modeling approach was developed, incorporating artificially prefabricated asperities along the sliding interface. Using 3D laser scanning to monitor slope deformation, the physical experiments provide the first direct evidence linking asperity rupture to landslide initiation. The interfacial asperity theory proves particularly effective in analyzing the three recurrent landslides along the Hushuo Expressway (2012–2017), where conventional engineering treatments failed, as all cases exhibited characteristic interfacial sliding mechanisms. The results reveal the nonlinear slip mechanisms of interfacial landslides: the first failure was controlled by asperities at the slope toe, the second by asperities at the slope crest, and the third by structural asperities. Due to post-failure excavation at the site, numerical simulations were employed to reconstruct the failure process, successfully reproducing the controlling effects of different asperity types on landslide evolution. The study also proposes the concept of targeted monitoring and early warning for interfacial landslides.The findings offer a novel theoretical perspective for understanding the progressive failure mechanisms of interfacial landslides and provide critical insights for the design of landslide mitigation measures.

## 1 Introduction

Interfacial landslides are among the most prevalent and frequently occurring landslide types on the China Loess Plateau, leading to significant economic and environmental losses [1–2]. These landslides pose a substantial threat to the sustainable development of local economies, particularly in regions where infrastructure projects are rapidly expanding [3].

Based on the classification proposed by Derbyshire [4], interfacial landslides in Northwest China can be categorized into four types, depending on the soil composition and the location of the failure plane: bedrock contact landslides, paleosol contact landslides, mixed landslides, and slides within loess. These landslides typically involve the deformation of soil layers along an underlying sliding zone. Due to their occurrence in relatively gentle terrains and their short sliding distances [5], interfacial landslides are often challenging to detect and are highly susceptible to disturbances from construction activities [6]. Extensive experimental research has demonstrated that the triggering mechanisms of these landslides are closely associated with surface water infiltration or rising groundwater levels [7,8]. Under these conditions, the sliding zone soil undergoes softening, leading to a reduction in shear strength and eventual landslide instability [9]. In recent years, the increase in infrastructure projects such as road construction, water management systems, and mining activities in Inner Mongolia has exacerbated the risk of landslides, particularly due to excavation, slope cutting, and heap loading. However, the lack of comprehensive theoretical models and advanced field-monitoring techniques has hindered effective landslide prediction and management.

The failure mechanisms of interfacial landslides are inherently complex and cannot be adequately explained by traditional linear mechanical models [10]. As a result, multi-scale and non-linear approaches are essential for understanding these phenomena. Recent studies by Huang, Qin, Xue et al., and Tang have focused on the critical components of landslide sliding zones, introducing the concept of “locking sections” that control landslide initiation. These studies have classified landslide sliding types into several categories, including three-stage sliding, retaining wall failure, block sliding, and multi-stage linear sliding, among others. The asperity theory, which shares similarities with the locking section theory, emphasizes the role of well-defined sliding zones, particularly at soil-rock interfaces [11–13].

Advancements in computational technology have significantly enhanced the application of numerical simulation in geotechnical engineering. Commonly used software such as FLAC, PFC, and Midas FEA employ various computational methods, including the finite element method, finite difference method, and distinct element method. These tools have been widely applied in the stability analysis of landslides, tunnels, and foundation pits. For instance, Liu, Xie et al. utilized PFC2D to simulate the failure modes of highway tunnels, examining the evolution of tunnel excavation failures and analyzing the mechanisms of shear failure at different depths [14]. Di et al. evaluated the horizontal and vertical deformations of railway subgrades induced by shield tunneling through numerical simulations [15]. Similarly, Wu, He et al. employed Midas GTS NX to model the Chaqishan ancient landslide, assessing the impact of various excavation processes on slope stability [16].

The development of landslide theories has also improved the accuracy of monitoring and early warning systems. Geotechnical monitoring equipment techniques can be broadly divided into contact and non-contact methods. While non-contact methods, such as GPS, InSAR, and 3D laser scanning, are effective for surface monitoring, internal data collection still relies on contact sensors [17–19]. Emerging technologies, including distributed optical fiber deformation monitoring, have further enhanced the precision of landslide monitoring [20]. However, the successful application of these advanced techniques depends on the availability of accurate theoretical models that can elucidate the underlying mechanisms of interfacial landslides.

## 2 Mechanical response test of asperity displacement and landslide deformation

### 2.1 Principle of asperity theory

Based on field observations and monitoring, the interfacial landslide development of the sliding zone is a non-linear process. According to classical landslide analysis, under uniform interfacial slip conditions, when the sliding zone front is subject to excess stress, slope acceleration decreases, no other external force influences the landslide, and the landslide will not stop. However, according to field observations, the landslide is non-linear and discontinuous, and there is creep deformation. As a single linear sliding mechanism system cannot be used to describe the whole process, it is therefore important to develop a progressive mechanism to describe the landslide process as a whole within the existing framework.

**Fig 1:**
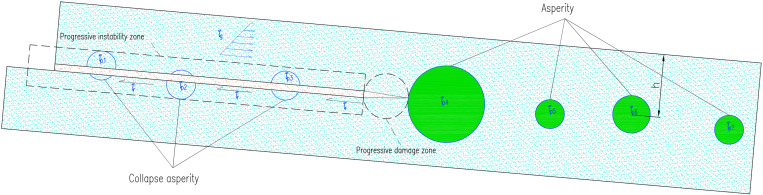
Failure mode of asperity in a landslide interface.

The maximum shear strength of an interfacial landslide is different at different positions in the sliding zone [21]. In the process of landslide instability and sliding, there are areas with high shear strength in the sliding zone, and the landslide will stop when the resistance is greater than the sliding force. In the process of reaching equilibrium, when the shear strength decreases or the landslide force increases due to other factors in areas with higher strength, the landslide will slide again until it meets the next area with higher shear strength. This is the basic view of asperity theory relating to interfacial landslides (Fig 1).

Asperity in an interfacial landslide is a concept that includes the part of the sliding zone with high strength or high friction force. The asperity can be composed of natural soil or an artificial structure, and its main role is to hinder further development of the landslide. Owing to the diversity of friction forms, the established equilibrium equations are different in each case, but the basic theories are those of force balance, yield criterion, and energy balance [22].

In Fig 1, where τg is the stress of slide, τr is the residual shear strength, τpi is the shear strength of the ith asperity.

Through analysis of the interfacial landslide according to asperity theory, during the sliding process, the interaction between layers that slide relative to each other in sliding zone is no longer linear. Considering the influence of other external factors such as rainfall, asperity can be summarized into three categories. In asperities with unstable residual shear stress (type I), the main features are that the sliding zone sliding force (τg − τr) is unstable, and, due to the action of the sliding belt water and interactions with the soil, residual shear strength τr is also unstable [23]. Although such asperities are not real, they can be considered widespread parts of the sliding zone that have slightly higher strength than the surrounding soil. Material-type asperities (type Ⅱ) are those with higher strength and shear strength than the surrounding soil body. Structural asperities (type Ⅲ) are generally structures with artificial skid resistance; their strength is much higher than that of the surrounding soil, and generally no damage will occur.

### 2.2 Scaled model test using similar materials

This study employs a scaled model test using similar materials to simulate the geological conditions of an actual interface landslide, investigating the influence of asperities within the sliding interface on slope movement. During the experiment, high shear-strength soil (asperities) was artificially created, and 3D laser scanning technology was utilized to continuously monitor surface deformation. By controlling the sequential failure of asperities, their impact on slope movement and surface deformation was observed, thereby validating and refining the asperity theory of interface landslides.

**Fig 2:**
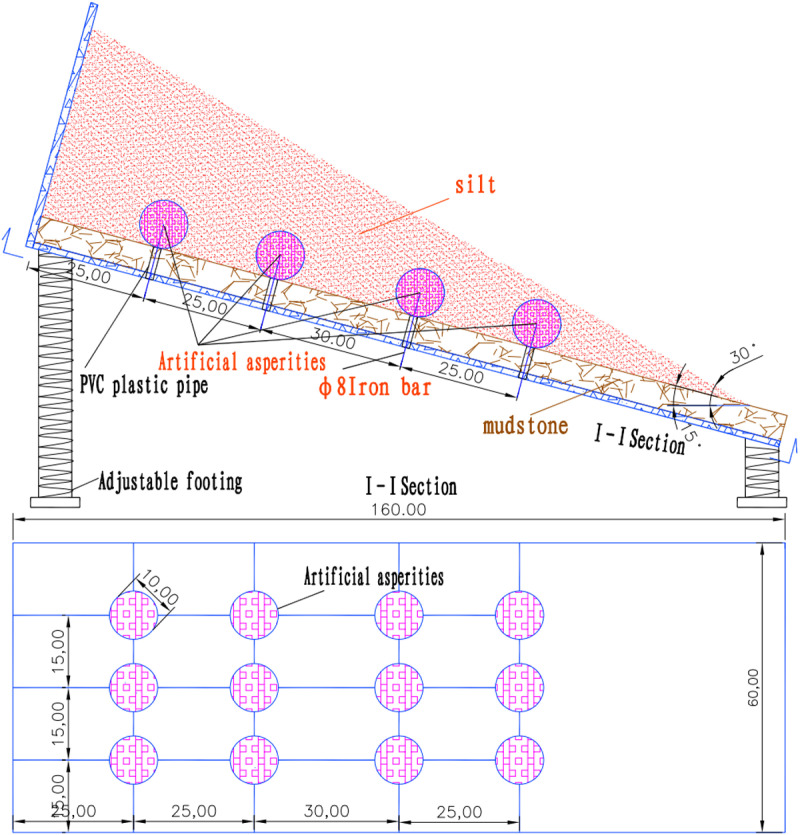
Schematic surface of scale model of similar material.

Based on actual landslide conditions, a scaled model measuring 150 cm × 60 cm × 50 cm was designed, with 12 artificial asperities embedded in the sliding interface to provide anti-sliding force and maintain the model in a limit equilibrium state (Fig 2). The experiment was divided into two groups: the first simulated a traction-type landslide by sequentially failing asperities from the bottom upward, recording surface deformation; the second simulated a thrust-type landslide by sequentially failing asperities from the top downward, analyzing the impact of asperity failure at different locations on surface deformation.

**Fig 3:**
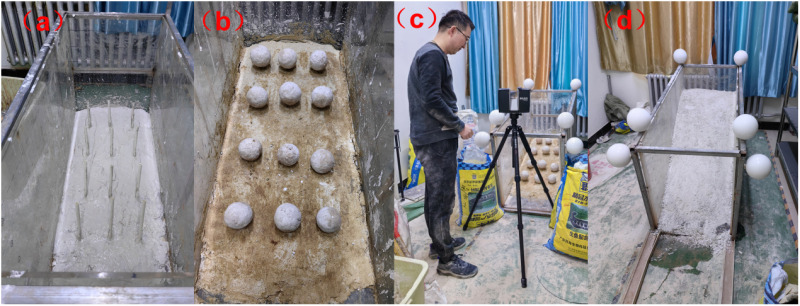
Photographs of the similar material scale model tests.

During the experiment, similar materials (mudstone) were laid and cured for two weeks to ensure stability. Asperities were controlled using PVC pipes and steel bars, and surface deformation was recorded using a 3D laser scanner. The results demonstrate that the sequence of asperity failure significantly influences the sliding mode and surface deformation of the slope, validating the engineering application value of the asperity theory of interface landslides (Fig 3).

**Fig 4:**
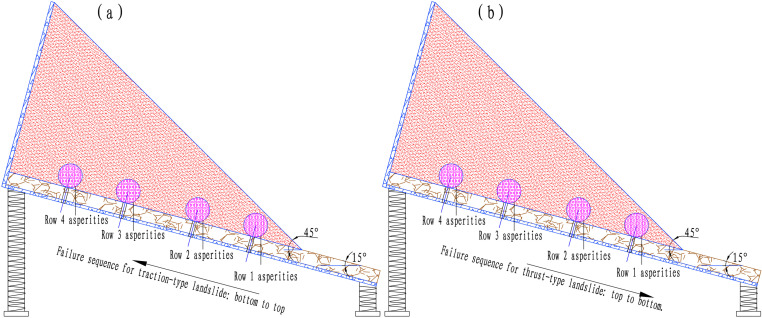
Failure sequence diagram of landslide asperity.

The experiment selected similar materials based on field geological data from landslides in Inner Mongolia. The overlying soil in most landslides consists of weak materials such as silty sand, clay, and silty clay. The bedrock was simulated as mudstone. Most interface landslides in Inner Mongolia occur at depths of 10–15 m. Given the maximum height of the model box (50 cm), the geometric similarity ratio for this experiment was set at 1:30. The materials simulated in this scaled model test include silty soil and mudstone, the composition and ratios are provided in the table below:

The purpose of the scaled model test using similar materials was to analyze the impact of asperity failure on slope surface deformation. Therefore, 3D laser scanning was conducted after each row of asperities failed, resulting in six datasets. For the traction-type landslide (Fig 4a), the scans included: (1) the initial distribution of asperities on the sliding interface; (2) the slope surface after soil overlay; (3) deformation after the failure of the first row of asperities; (4) deformation after the failure of the second row; (5) deformation after the failure of the third row; and (6) deformation after the failure of the fourth row. During the experiment, as the first to fourth rows of asperities failed, numerous cracks formed on the slope surface, which were captured using 3D laser scanning. For the thrust-type landslide, the sequence of asperity failure was reversed, as illustrated in Fig 4b.

### 2.3 Results of scaled model tests with similar materials

**Fig 5:**
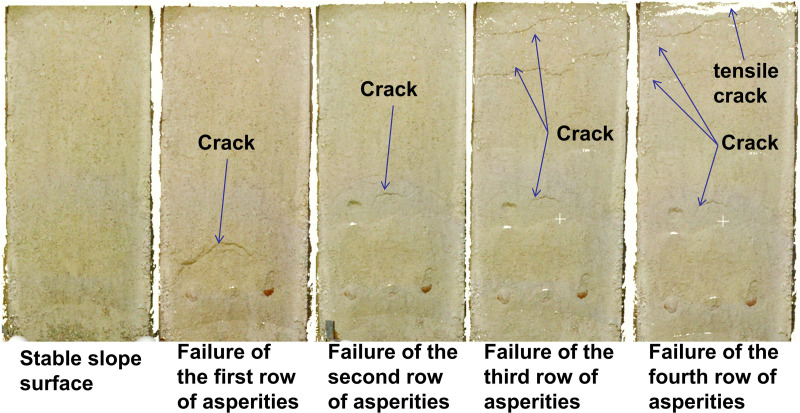
3D laser scanning image of a traction-type landslide slope.

**Fig 6:**
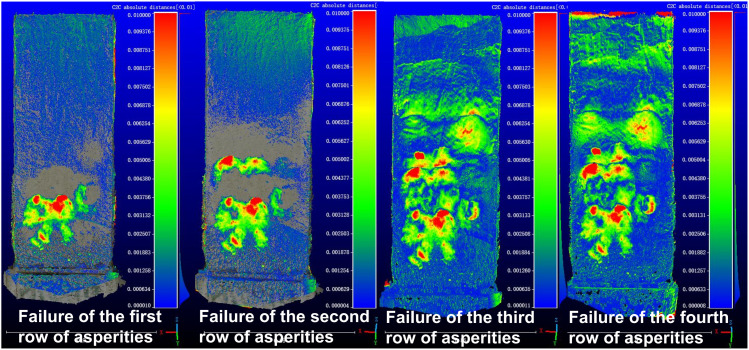
Point cloud comparison of retrogressive (traction-type) landslide.

In scaled model tests of traction-type and thrust-type landslides using similar materials, 3D laser scanning was employed to acquire point cloud data, which were then analyzed through collision simulation in CloudCompare software. Using the original slope surface as a reference plane, the morphological changes at four different failure stages were systematically compared with the reference plane to investigate their deformation evolution patterns. The morphological characteristics and point cloud collision simulation results of the traction-type landslide at each stage are presented in Figs 5 and 6.

The retrogressive landslide development was characterized by sequential failures of asperity rows. The first asperity row failure generated chair-shaped tension cracks, with the scarp developing at the front edge of the second asperity row. When the second asperity row failed, new cracks formed behind this row, causing downward sliding that buried the cracks from the first row failure. Failure of the third asperity row produced two additional cracks at the slope rear. The final failure of the fourth asperity row mainly widened existing cracks without creating new fractures due to confinement by the test chamber wall, only increasing the gap between the slope rear and chamber wall.

Point cloud comparisons revealed distinct deformation patterns for each failure stage. The first asperity row failure caused severe deformation in its overlying soil. The second row failure also induced significant deformation, though less intense than the first row but affecting a broader area including the rear slope. The third row failure showed reduced deformation intensity but further expanded influence, forming a continuous sliding zone that led to overall slope displacement with tensile characteristics due to boundary effects. The fourth row failure resulted in complete slope failure and deposition. These observations demonstrate that asperity row failures dominantly control surface deformation, with thinner overburden corresponding to more intense but localized deformation, showing clear diffusion angle effects in influence propagation.

**Fig 7:**
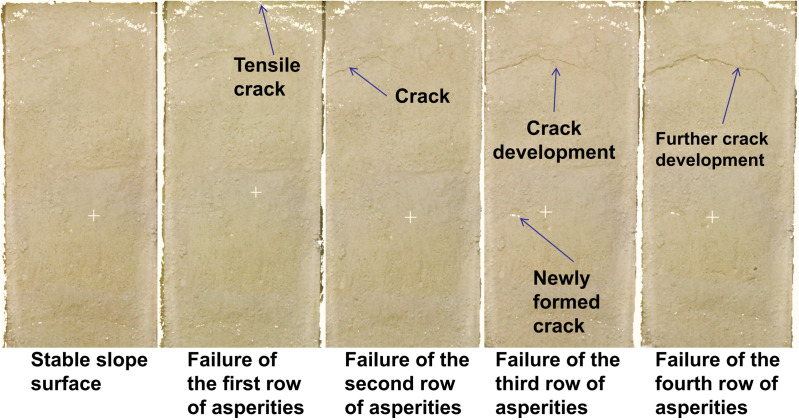
3D laser scanning image of a thrust-type landslide slope.

**Fig 8:**
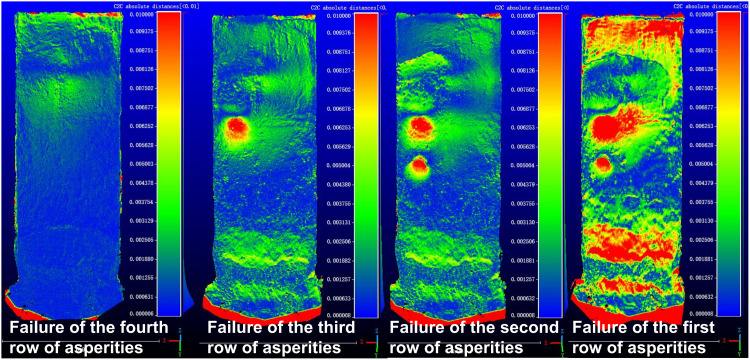
Point cloud comparison of retrogressive (thrust-type) landslide.

The thrust-type landslide results are shown in Figs 7 and 8. The experimental results demonstrate that these two landslide types exhibit significantly different deformation characteristics and failure mechanisms.

Initial observations of the thrust-type landslide at the planar scale revealed distinct deformation patterns during progressive asperity failures. When the fourth row of asperities failed, no significant slope deformation occurred due to insufficient thrust force from the limited rear soil mass, as the resisting force from basal asperities exceeded the applied thrust. Failure of the third row generated minor cracks with limited extension at the slope rear. The second row failure substantially reduced resistance, increasing the driving force of the overlying soil mass. With a well-developed sliding zone already formed during the third row failure, the upper soil mass displaced as a coherent block, widening pre-existing cracks. Final failure of the first row did not produce new fractures but further extended rear-slope cracks under accumulating driving forces.

Point cloud data demonstrated fundamentally different deformation behavior compared to retrogressive landslides. The fourth row failure induced only minor widespread displacement at the slope rear. Third row failure caused intense deformation in overlying soil, with clear toe accumulation and significant overall slope displacement. Second row failure resulted in localized deformation that advanced the sliding mass. First row failure triggered catastrophic deformation as the entire driving force concentrated on toe asperities.

Scaled physical modeling confirmed a direct correlation between asperity failure at the sliding interface and surface deformation. Surface deformation features effectively indicate asperity positions along the sliding plane. This study highlights the downslope-propagating failure mechanism unique to thrust-type landslides, providing critical insights for optimizing support structures in landslide mitigation engineering. The distinct “top-down” progression contrasts sharply with the “bottom-up” failure mode of retrogressive landslides.

## 3 Overview of the Hushuo Expressway

**Fig 9:**
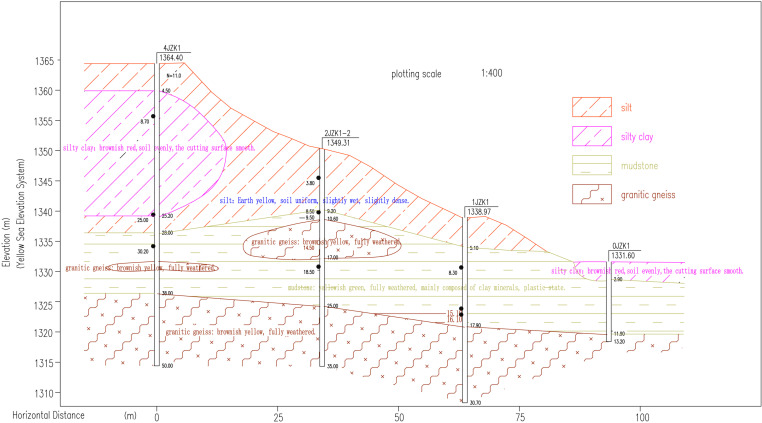
Engineering geology profile of the landslide case segment.

The Hushuo Expressway is an important expressway in Inner Mongolia, as it is an important truck route connecting North and Northwest China. Construction has not only provided another fast route into Beijing but also effectively diverted traffic from the Beijing–Tibet Expressway.

landslide location along the Hohhot-Shahukou Expressway at K85. Fig 9 presents the geological profile consisting of upper Quaternary silt and silty clay overlying Lower Cretaceous completely weathered mudstone and granite gneiss.

The area is situated at approximately 1300m elevation with a typical continental climate characterized by aridity, frequent winds, and concentrated summer precipitation. Hydrogeological conditions feature a large surface catchment area and deep scouring gullies. No perennial surface water or groundwater was detected within drilling depth. Precipitation infiltration through Tertiary clay and Cretaceous mudstone layers increases soil water content while reducing shear strength. The landslide area has a peak ground acceleration of 0.15 g corresponding to seismic intensity VII.

### 3.1 Characteristics of landslides on the Hushuo Expressway

The landslide section is located at K85km on the Hohhot to Shahukou section of the Hushuo Expressway. From July 2012 to July 2017, two large-scale landslides and one creep deformation occurred. Fieldwork provides the basis and starting point for the research and analysis of engineering geological problems. By summarizing the deformation of the three landslides, the data generally support a mechanism of gradual friction creep mutation.

**Fig 10:**
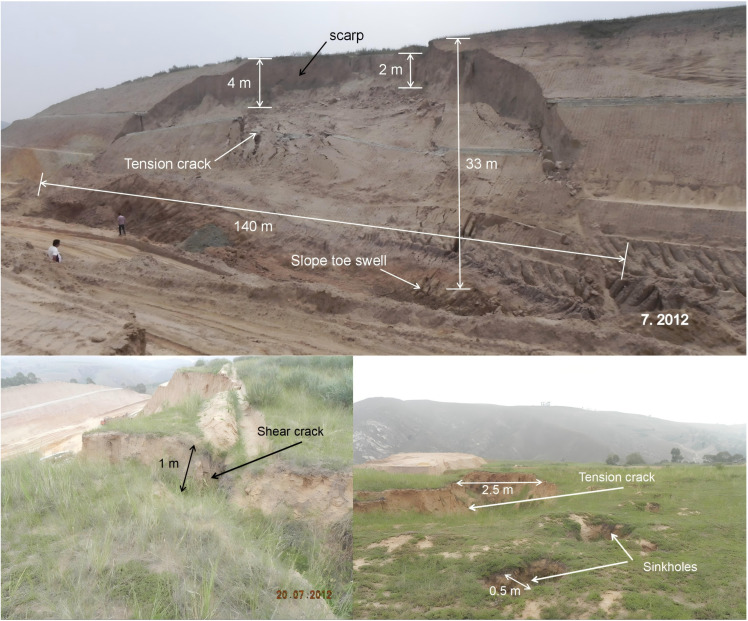
Photographs of the landslide in July 2012.

Due to unloading at the foot of the subgrade excavation, the slope slipped at K84 + 660-K84 + 800 in July 2012. According to field measurements, the main slide direction of the landslide body is appoximately NW15°–20°, the landslide is about 33 m high, the slope shape is concave to the soil slope, and the thickness of the slide body is about 5–15 m. The perimeter of the landslide is clear, the scarp behind the landslide is 2–4 m in height, and the western slope foot of the leading edge is partially raised (Fig 10).

**Fig 11:**
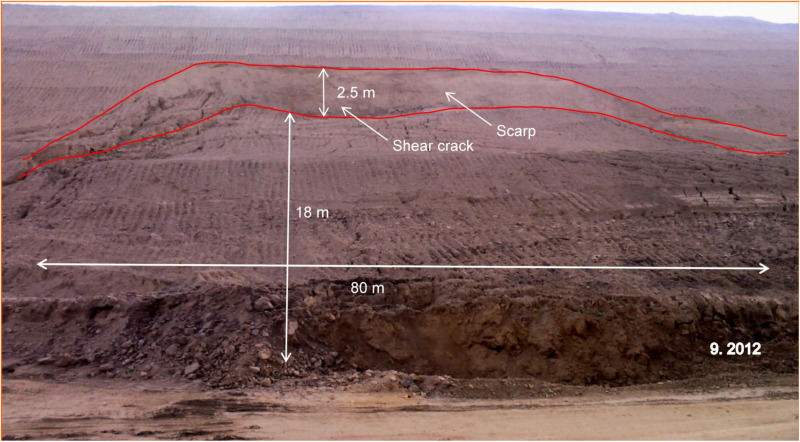
Photograph of the landslide in September 2012.

After the landslide occurred, the deformed slope and the structurally damaged soil layer were excavated to reduce the slope gradient. After the completion of unloading, the landslide occurred again in September 2012, with damage from position K84 + 700 to K84 + 780, and there were no tension cracks from the trailing edge to the top (Fig 11). After the second landslide, the surface of the slope was cleaned, micro piles were set on the slope, and cast-in-place piles were arranged at the foot of the slope. To prevent local soil collapse, mortar stone with a strength of 10 MPa was set in the section of slope collapse for slope protection.

**Fig 12:**
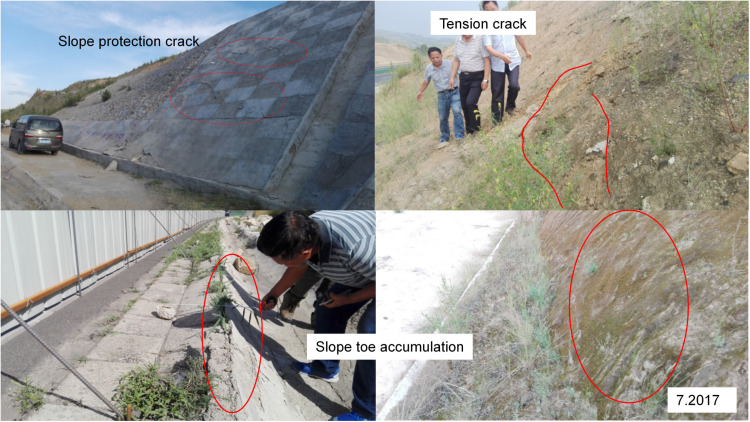
Photographs of creep deformation in July 2017.

In June 2017, the expressway’s maintenance department found that the protective wall had cracked, there were obvious cracks at the top, the soil had moved down at the foot of the slope, part of the soil had invaded the expressway land, and the slope showed characteristics of creep deformation (Fig 12). Treatment measures were mainly linked to prevent water seepage on the slope. A C25 concrete arch water-cut frame was used, and an inclined drainage pipe was set in the arch, leading to water seepage from the slope to the slope foot through the longitudinal drain pipe, and precipitation was discharged into the side ditch through the encrypted seepage hole.

### 3.2 Analysis via GPS monitoring

**Fig 13:**
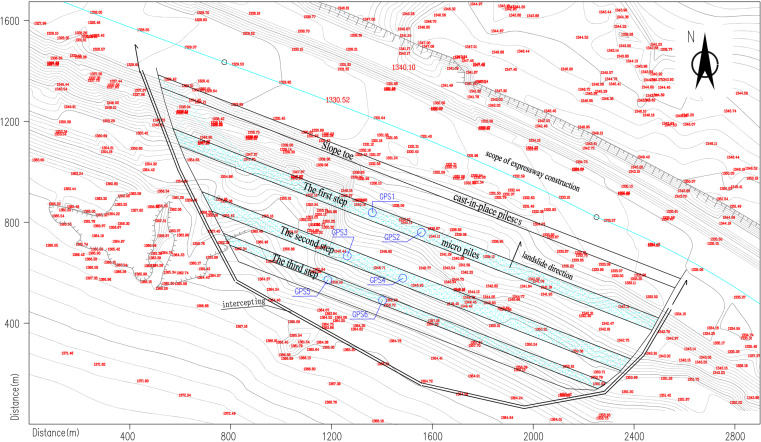
GPS monitoring layout.

After the second landslide, six GPS monitoring points (GPS1–GPS6) were deployed on the landslide to monitor the cumulative subsidence of the slope and the maximum subsidence rate from April 2013 to November 2013. The monitoring locations are shown in Fig 13.

During the monitoring period, cumulative subsidence was highest for GPS5 and GPS6, and the subsidence rate was highest in September due to high rainfall. Due to the low accuracy of the GPS devices, no data were measured when subsidence was low. According to the experimental data, the loess is relatively uniform, its permeability coefficient is low, and it takes a long time for rainwater to penetrate down from the surface. However, part of the loess landslide has widely distributed tensile cracks, sinkholes, and other structural cracks; and, under conditions of natural rainfall, surface water will infiltrate along the crack channel directly into the landslide body, and even directly along the sliding interface, decreasing soil strength and causing instability of the loess landslide.

## 4 Methods

Based on asperity theory of interface landslide, the numerical simulation software is used to simulate the actual slope sliding process to find the position of the asperity. The purpose of the study is to explain the mechanism of previous landslides by asperity theory, and to explore the application value of interface landslide asperity theory in practical engineering.

Because the actual landslide site has been excavated and unloaded, it is impossible to carry out field tests to analyze the landslide mechanism. The experimental method of this study mainly through numerical simulation software to simulates the Hushuo landslides. The soil mechanics data of landslides are collected, as shown in Tables 1–3. 

**Table 1:** Similar material ratio information.

Target Soil Type	Similar Material Composition	Mix Ratio (by Mass)	Remarks
Silty Clay	Detergent:Gypsum:Barite Powder:Quartz Sand:Bentonite:Water	7:13:51:129:40.47:29.32	
Mudstone	Iron Concentrate:Barite Powder:Quartz Sand:Gypsum Powder:Water:Glycerol	15.2:51:15.1:7.2:10:1.5	Cured for 7 days

**Table 2:** Soil test results Table 1.

	Distribution depth	Wet density	Field water content	Dry density	Porosity	Void ratio	Saturation	Liquid limit	Plastic limit
	m	ρ	w	ρ_d_	n	e_0_	Sr	W_L_	W_P_
		g/cm^3^	%	g/cm^3^	%		%	%	%
silt	0 ~ -5	1.90	16.6	1.63	39.6	0.657	68.2	25.7	21.7
	-5 ~ -18	1.90	16.8	1.63	39.8	0.660	68.7	25.6	21.5
	-18 ~ -35	1.94	24.4	1.56	42.2	0.731	90.1	25.5	21.6
silty clay	-5 ~ -25	1.89	27.2	1.49	45.2	0.824	89.5	38.8	25.2
mudstone	-8 ~ -35(Ⅰ)	1.89	36.0	1.39	49.3	0.972	100.0	51.2	24.4
	-8 ~ -35(Ⅱ)	1.81	26.6	1.43	47.2	0.896	80.5	38.7	25.3
	-8 ~ -35(Ⅲ)	2.07	21.5	1.70	37.8	0.608	96.8	39.6	22.0
	-8 ~ -35(Ⅳ)	1.94	26.6	1.53	44.1	0.788	92.5	58.8	25.5
Granite -gneiss	-25 ~ -60	1.85	28.2	1.44	47.3	0.899	86.0	50.7	24.8

**Table 3:** Soil test results Table 2.

	Distribution depth	plasticity index	liquidity index	compressibility	compression modulus	cohesion	internal friction angle
	m	I_P_	I_L_	a	E_S_	C	φ
				Mpa^-1^	Mpa	K_pa_	°
silt	0 ~ -5	4.0	-1.28	0.274	5.953	8.27	24.4
	-5 ~ -18	4.1	-1.15	0.19	8.599	15.03	30.1
	-18 ~ -35	3.9	0.72	0.314	5.435	14.75	25.9
silty clay	-5 ~ -25	13.6	0.15	0.215	8.496	45.45	20.5
mudstone	-8 ~ -35(Ⅰ)	26.8	0.43	0.151	13.032	59.75	12.5
	-8 ~ -35(Ⅱ)	13.4	0.10	0.601	3.164	76.8	8.6
	-8 ~ -35(Ⅲ)	17.6	-0.03	0.151	10.591	153.2	15.7
	-8 ~ -35(Ⅳ)	33.3	0.03	0.139	12.83	40.3	23.7
Granite -gneiss	-25 ~ -60	25.9	0.13	0.181	10.515	56.3	13.6

Due to the limitations of geological exploration sampling, it is impossible to obtain the distribution of soil strength at the entire landslide through geotechnical test results. In the three landslide processes of Hushuo Expressway, the disturbance position is clear. Therefore, according to the actual sliding situation of the slope, when simulating the sliding process of the slope, it is necessary to adjust the soil characteristics at the sliding interface, and finally determine the distribution position of the soil with different strength in the sliding interface, and then the asperity positions can be obtained. Therefore, the numerical simulation test in this study is different from the conventional numerical simulation. It is a process of rehearsal and trial calculation, and multiple modeling is needed to achieve the effect of fitting the actual landslide sliding process. The finite element analysis for slope stability was performed using Midas FEA software. This approach iteratively reduces the shear strength parameters until numerical non-convergence indicates slope failure.

## 5 Results

**Fig 14:**
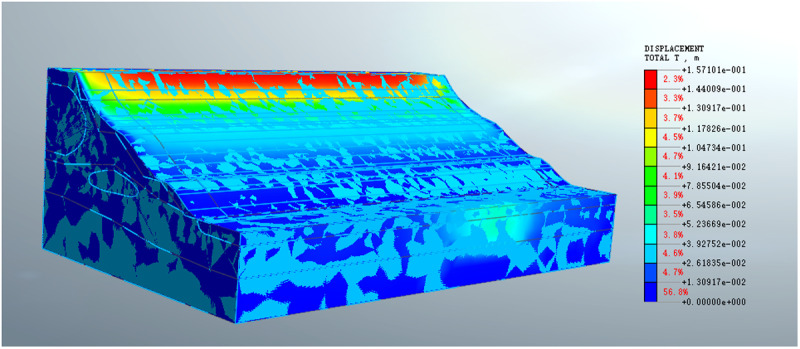
Numerical simulation displacement of undisturbed slope.

The strength reduction method is used for numerical simulation, and the stability of the slope before the disturbance is checked, the safety factor is 1.16. Before the toe of the slope was excavated, the slope body was relatively stable, and only a small displacement occurs on the top surface of the slope, as shown in Fig 14.

### 5.1 First landslide simulation

The first landslide inducement is that the soil at the toe of the slope is excavated. At first numerical simulation divides the same kind of soil into a whole for meshing. The calculation results show that the soil is stable and fails to reflect the sliding process of the actual landslide. Based on the theory of interfacial landslide asperity theory, the soil at the sliding interface is finely divided, and the soil properties and parameters are defined for the same soil at different positions, and multiple trial simulations are carried out.

**Fig 15:**
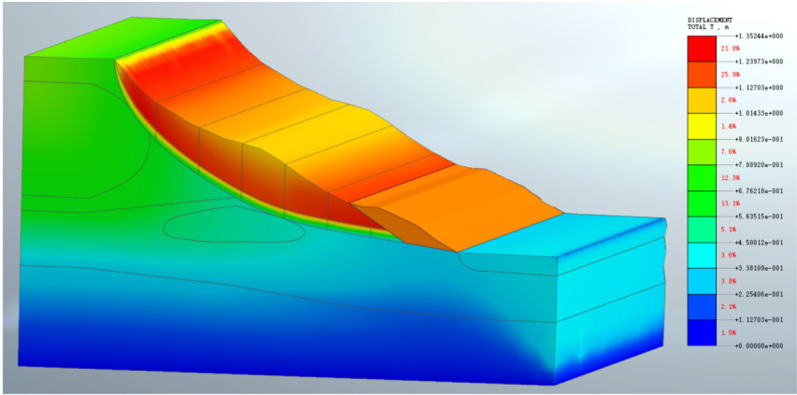
The first landslide simulation displacement diagram.

**Fig 16:**
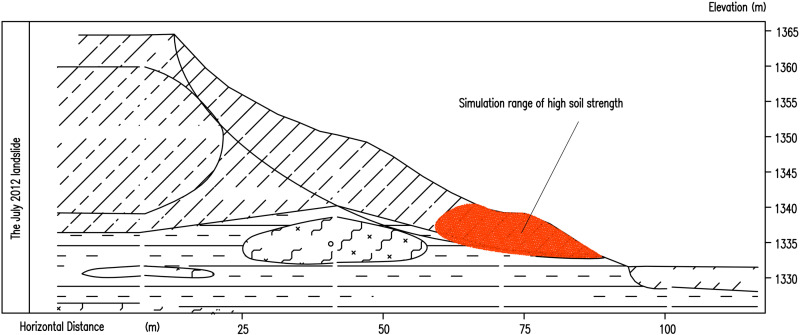
The first landslide numerical simulation of high strength range diagram.

Finally, the results consistent with the actual landslide displacement are obtained as shown in Fig 15. In the simulation process, the defined high-strength soil (asperity) is shown in Fig 16.

### 5.2 Second landslide simulation

**Fig 17:**
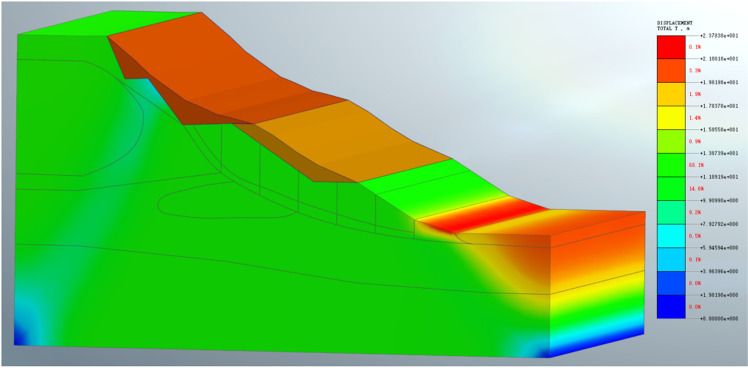
The second landslide simulation displacement diagram.

**Fig 18:**
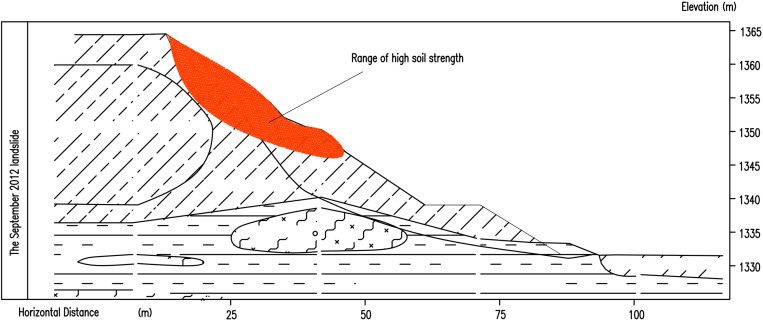
The second landslide numerical simulation of high strength range diagram.

The second landslide occured after the slope top was unloaded. According to the actual landslide condition, the parameters of each part of the soil are adjusted. Although the soil parameters are adjusted many times, it is still impossible to simulate the displacement of the actual engineering slope.The simulated displacement is shown in Fig 17. After analysis, there are two reasons for this situation. The first is that the strength reduction method is used in the numerical simulation, and the tensile stress of the soil is not considered. The soil with higher strength is located at the top of the slope and pulls the soil below. After unloading, the soil at the top of the slope is destroyed and slides. The second point is that there is water accumulation in the second step, which infiltrates down the step, softening the soil in the sliding zone and causing landslide. The position of the high-strength soil (asperity) is shown in Fig 18.

After the second landslide, due to the addition of protective measures such as anti-slide piles, the third landslide only shows the characteristics of creeping, so there is no need to carry out numerical simulation of the third landslide, and the anti-slide pile can be clearly defined as a structural asperity.

## 6 Discussion

### 6.1 Interfacial landslides

According to geological surveys, the landslide zone here comprises silt–mudstone, which is one of the most widely distributed and most frequently occurring interface landslide types on the China Loess Plateau. Research of such interface landslides is also a hotspot in the engineering geological field in China. According to the analysis of the field situation at K85 of the Hushuo Expressway, gray–green, fully weathered mudstone is distributed in the lower part of the slope. The mudstone layer shows weak–strong expansion, soft expansion under high humidity, shrinkage due to water loss, repeated expansion, and poor engineering characteristics. Vertical pore development of the upper silt makes it highly permeable, and the lower clay and mudstone are weakly permeable. Under atmospheric precipitation, the water content of the lower soil has adverse effects, causing the sliding zone to soften [24].

### 6.2 Engineering failure analysis by asperity theory of interfacial landslides

The unique “double layer heterogeneous” slope structure of the silt–mudstone means that the contact surface between the silt and the mudstone represents a weak zone, and may easily form the landslide slip zone and control movement of the landslide.

**Fig 19:**
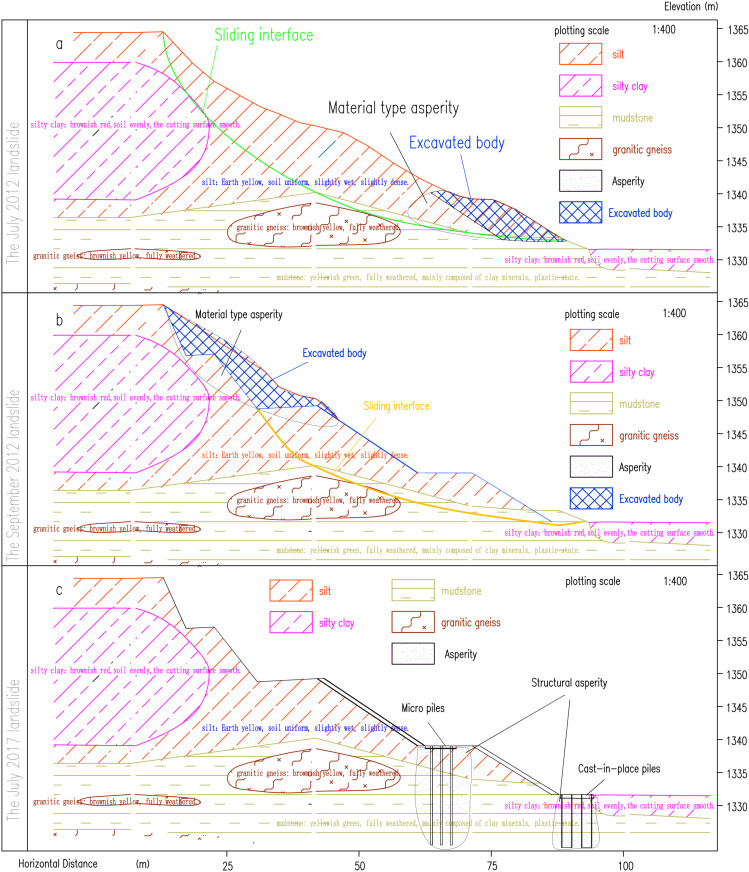
Sketches of the three landslides.

A ccording to the results of numerical simulation, the following conclusions can be obtained. The landslide occurred in July 2012. Material-type asperity was located at the foot of the slope. Excavation of the roadbed destroyed the asperity and changed the state of slope stability and landslide instability along the mudstone strata and the upper soil interface slide; therefore, the landslide is a shallow traction soil slide (Fig 19a). In the process of slope instability and sliding, a new material-type asperity was generated at the top of the slope. After landslide unloading, the new asperity was destroyed, the slope failed to reach an equilibrium state (Fig 19b), and a further landslide occurred in September 2012. From September 2012 to June 2017, water penetrated into the zone of sliding, the sliding interface gradually softened, and the shear strength in the area of stress concentration gradually decreased. In June 2017, due to landslide treatment measures, numerous structural asperities were added to the landslide slope manifesting only as creep deformation (Fig 19c), leading to the third landslide.

In the process of numerical simulation, the second landslide cannot be reproduced, which indicates that the conventional limit equilibrium theory cannot explain all the landslide phenomena, and the asperity theory makes up for the vacancy. The asperity theory of interfacial landslides can provide theoretical guidance for field investigations of landslides at an early stage, quickly and accurately identify the current state of interfacial landslides, and provide the necessary basic theoretical support for the implementation of subsequent monitoring and early warning systems, and engineering prevention of future landslides. Therefore, the asperity theory of interfacial landslides represents a theoretical innovation, and an improvement and reprocessing of the existing knowledge system.

### 6.3 Targeted monitoring based on asperity theory

Landslide monitoring methods and equipment vary significantly in size, working principles, complexity, and cost, requiring selection based on specific conditions. For interfacial landslides, the choice should consider geographic environment, available equipment, monitoring duration, precision requirements, and climatic/geological factors. 3D laser scanning and InSAR represent optimal solutions.

Through asperity theory, the position of the asperity in the sliding interface can be related to the landslide surface displacement, and the key positions (targets) of the landslide surface can then be targeted, which can improve the accuracy of monitoring and reduce the input of monitoring equipment. In the process of monitoring interface landslide deformation, because 3D laser scanning and InSAR are active scanning equipment, real-time landslide warning is not available. Therefore, after multiple scans to determine landslide surface targets, monitoring at a small range but high precision via passive equipment monitoring of targets should be implemented as a new interface landslide monitoring method.

The new monitoring scheme was implemented on the Hushuo high-speed landslide, initiating prior to slope movement. Initial monitoring focused on the slope toe (asperity location) and crest (maximum settlement area), requiring only two GPS points (GPS5–6). Following the first landslide, which caused top-slope unloading, monitoring shifted to the new asperity zone at the slope top (GPS3–4). After the second landslide, structural asperities induced creep deformation with whole-slope accumulation, making crest settlement (GPS5–6) the most significant indicator. This targeted approach reduced monitoring costs while improving accuracy, as illustrated in Fig 13.

## 7 Conclusions

Based on the analysis of field deformation characteristics of an historical landslide on the Hushuo Expressway, asperity theory proved to be of value, and the theory of interface landslides was enriched. Asperity theory provides a more efficient scheme for interface landslide monitoring and engineering hazard prevention. This study has the following conclusions:

1. A novel scaled physical modeling method was established to simulate interfacial landslides through artificial asperity embedding along sliding interfaces. Controlled failure experiments of interfacial asperities, combined with three-dimensional point cloud analysis, confirmed the direct control of asperity rupture on surface deformation patterns. The study further identified fundamentally opposing deformation mechanisms between traction-type and thrust-type landslides.
2. For the first time, asperity theory is used to describe the whole process of field landslide, making the analysis more suitable for the actual process of mountain sliding.The interfacial asperity theory, integrated with numerical simulations, was employed to analyze the multi-phase deformation mechanisms of the Hushuo Expressway landslide. The analysis revealed distinct asperity distributions during successive failure events: initial failure was controlled by basal asperities at the slope toe, while subsequent failure was governed by crown asperities at the slope crest.
3. The feasibility of using asperity theory for targeted monitoring of landslides is discussed; it can improve the precision of monitoring and reduce monitoring investment.The monitoring protocol initiated with comprehensive slope surface analysis using 3D laser scanning and InSAR to identify potential asperity locations along the sliding interface based on surface deformation patterns. Targeted GNSS monitoring points were subsequently deployed at these critical asperity zones, establishing an optimized interfacial landslide monitoring system that combines regional surface surveillance with localized displacement measurements.

## References

[1] Liu Z, Liu F, Ma F, Wang M, Bai X, Zheng Y, et al. Collapsibility, composition, and microstructure of loess in China. Can Geotech J. 2016;53(4):673–86. doi: 10.1139/cgj-2015-0285

[2] Xie X, Qi S, Zhao F, Wang D. Creep behavior and the microstructural evolution of loess-like soil from Xi’an area, China. Eng Geol. 2018;236:43–59.

[3] Peng J, Wang S, Wang Q, Zhuang J, Huang W, Zhu X, et al. Distribution and genetic types of loess landslides in China. J Asian Earth Sci. 2019;170:329–50. doi: 10.1016/j.jseaes.2018.11.015

[4] Derbyshire E, Meng XM. Landslides in the thick loess terrain of North-west China. Eng Geol. 2001;59:201–2.

[5] Kimura S, Nakamura S, Vithana SB. Influence of effective normal stress in the measurement of fully softened strength in different origin landslide soils. Soil Tillage Res. 2015;145:47–54. doi: 10.1016/j.still.2014.07.018

[6] Li S, Li C, Yao D, Liu C. Interdisciplinary asperity theory to analyze nonlinear motion of loess landslides with weak sliding interface. Landslides. 2020;17(12):2957–65. doi: 10.1007/s10346-020-01479-3

[7] Ávila FF, Alvalá RC, Mendes RM, Amore DJ. The influence of land use/land cover variability and rainfall intensity in triggering landslides: a back-analysis study via physically based models. Nat Hazards. 2020;105(1):1139–61. doi: 10.1007/s11069-020-04324-x

[8] Chen G, Meng X, Qiao L, Zhang Y, Wang S. Response of a loess landslide to rainfall: observations from a field artificial rainfall experiment in Bailong River Basin, China. Landslides. 2017;15(5):895–911. doi: 10.1007/s10346-017-0924-6

[9] Wang J, Zhang D, Wang N, Gu T. Mechanisms of wetting-induced loess slope failures. Landslides. 2019;16(5):937–53. doi: 10.1007/s10346-019-01144-4

[10] Xu L, Dai FC, Tham LG, Tu XB, Min H, Zhou YF, et al. Field testing of irrigation effects on the stability of a cliff edge in loess, North-west China. Eng Geol. 2011;120(1–4):10–7. doi: 10.1016/j.enggeo.2011.03.007

[11] Huang R, Chen G, Tang P. Precursor information of locking segment landslides based on transient characteristics. CJRME. 2017;36(3):521–33. doi: 10.13722/j.cnki.jrme.2016.1100

[12] Xue L, Qin SQ, Pan XH, Chen HR, Yang BC, Zhang K. Mechanism and physical prediction model of instability of the locked-segment type slopes. J Eng Geol. 2018;26(1):179–92. doi: 10.13544/j.cnki.jeg.2018.01.020

[13] Tang H. Advance and prospects of major landslides prediction and forecasting. Bull Geol Sci Technol. 2022;41(6):1–13.

[14] Liu T, Xie Y, Feng ZH, Luo YB, Wang K, Xu W. Better understanding the failure modes of tunnels excavated in the boulder-cobble mixed strata by distinct element method. Eng Fail Anal. 2020;116:104712. doi: 10.1016/j.engfailanal.2020.104712

[15] Di H, He P, Li X, Xiao F, Chen H. Influence of large-diameter shield tunneling on deformation of adjacent high-speed railway subgrade in soft soils and effectiveness of protective measures. Tunnelling Underground Space Technol. 2025;156:106260. doi: 10.1016/j.tust.2024.106260

[16] Wu L, He K, Guo L, Zhang J, Sun L, Jia Y. Research on the excavation stability evaluation method of Chaqishan ancient landslide in China. Engineering Failure Analysis. 2022;141:106664. doi: 10.1016/j.engfailanal.2022.106664

[17] Rosi A, Tofani V, Tanteri L, Tacconi Stefanelli C, Agostini A, Catani F, et al. The new landslide inventory of Tuscany (Italy) updated with PS-InSAR: geomorphological features and landslide distribution. Landslides. 2017;15(1):5–19. doi: 10.1007/s10346-017-0861-4

[18] Di H, Zhou S, Xiao J, Gong Q, Luo Z. Investigation of the long-term settlement of a cut-and-cover metro tunnel in a soft deposit. Eng Geol. 2016;204:33–40. doi: 10.1016/j.enggeo.2016.01.016

[19] Alimohammadlou Y, Tanyu BF, Abbaspour A, Delamater PL. Automated landslide detection model to delineate the extent of existing landslides. Nat Hazards. 2021;107(2):1639–56. doi: 10.1007/s11069-021-04650-8

[20] Kogure T, Okuda Y. Monitoring the vertical distribution of rainfall‐induced strain changes in a landslide measured by distributed fiber optic sensing with Rayleigh backscattering. Geophysical Research Letters. 2018;45(9):4033–40. doi: 10.1029/2018gl077607

[21] Zhuang J, Peng J, Wang G, Javed I, Wang Y, Li W. Distribution and characteristics of landslide in Loess Plateau: a case study in Shaanxi province. Eng Geol. 2018;236:89–96. doi: 10.1016/j.enggeo.2017.03.001

[22] Li S, Li C, Yao D, Liu C, Zhang Y. Multiscale nonlinear analysis of failure mechanism of loess-mudstone landslide. Catena. 2022;213:106188. doi: 10.1016/j.catena.2022.106188

[23] Springman SM, Thielen A, Kienzler P, Friedel S. A long-term field study for the investigation of rainfall-induced landslides. Géotechnique. 2013;63(14):1177–93. doi: 10.1680/geot.11.p.142

[24] Xu J, Wang Z, Ren J, Wang S, Jin L. Mechanism of slope failure in loess terrains during spring thawing. J Mt Sci. 2018;15(4):845–58. doi: 10.1007/s11629-017-4584-8

